# Potential of dietary hemp and cannabinoids to modulate immune response to enhance health and performance in animals: opportunities and challenges

**DOI:** 10.3389/fimmu.2023.1285052

**Published:** 2023-12-04

**Authors:** Faiz-ul Hassan, Chunjie Liu, Maryam Mehboob, Rana Muhammad Bilal, Muhammad Asif Arain, Faisal Siddique, Fengming Chen, Yuying Li, Jingmeng Zhang, Pengjun Shi, Biguang Lv, Qian Lin

**Affiliations:** ^1^ Institute of Bast Fiber Crops, Chinese Academy of Agricultural Sciences, Changsha, China; ^2^ Faculty of Animal Production and Technology, Cholistan University of Veterinary and Animal Sciences, Bahawalpur, Pakistan; ^3^ Department of Zoology, Wildlife and Fisheries, University of Agriculture, Faisalabad, Pakistan; ^4^ Faculty of Veterinary and Animal Sciences, Lasbela University of Agriculture, Water and Marine Sciences, Uthal, Balochistan, Pakistan; ^5^ Hunan Provincial Key Laboratory of the TCM Agricultural Biogenomics, Changsha Medical University, Changsha, China

**Keywords:** animals, cannabinoids, hemp seed, therapeutic potential, anti-oxidant, immunomodulation

## Abstract

Cannabinoids are a group of bioactive compounds abundantly present in *Cannabis sativa* plant. The active components of cannabis with therapeutic potential are known as cannabinoids. Cannabinoids are divided into three groups: plant-derived cannabinoids (phytocannabinoids), endogenous cannabinoids (endocannabinoids), and synthetic cannabinoids. These compounds play a crucial role in the regulation various physiological processes including the immune modulation by interacting with the endocannabinoid system (A complex cell-signaling system). Cannabinoid receptor type 1 (CB1) stimulates the binding of orexigenic peptides and inhibits the attachment of anorexigenic proteins to hypothalamic neurons in mammals, increasing food intake. Digestibility is unaffected by the presence of any cannabinoids in hemp stubble. Endogenous cannabinoids are also important for the peripheral control of lipid processing in adipose tissue, in addition to their role in the hypothalamus regulation of food intake. Regardless of the kind of synaptic connection or the length of the transmission, endocannabinoids play a crucial role in inhibiting synaptic transmission through a number of mechanisms. Cannabidiol (CBD) mainly influences redox equilibrium through intrinsic mechanisms. Useful effects of cannabinoids in animals have been mentioned e.g., for disorders of the cardiovascular system, pain treatment, disorders of the respiratory system or metabolic disorders. Dietary supplementation of cannabinoids has shown positive effects on health, growth and production performance of small and large animals. Animal fed diet supplemented with hemp seeds (180 g/day) or hemp seed cake (143 g/kg DM) had achieved batter performance without any detrimental effects. But the higher level of hemp or cannabinoid supplementation suppress immune functions and reduce productive performance. With an emphasis on the poultry and ruminants, this review aims to highlight the properties of cannabinoids and their derivatives as well as their significance as a potential feed additive in their diets to improve the immune status and health performance of animals.

## Highlights

➢ Cannabinoids are a class of naturally occurring compounds found in the cannabis plant that plays a crucial role in regulating various physiological processes, in animals.
➢ Dietary cannabinoids could play a role, in improving appetite regulation, reducing inflammation, managing stress, and promoting overall well-being in animals.
➢ Certain cannabinoids, such as CBD (cannabidiol), might have the potential to improve feed efficiency in animals.
➢ Modulating the rumen microbiome through cannabinoids might have broader implications for gut health in ruminant animals, influencing overall well-being and potentially reducing the risk of digestive disorders.
➢ Dietary cannabinoids serve as a natural alternative to traditional animal health interventions, such as antibiotics or anti-inflammatories.
➢ Cannabinoids have been observed to exhibit protective effects against challenges posed by endotoxins and lipopolysaccharides.

## 1 Introduction

The current era of antibiotic resistance has raised concerns about the use of antibiotics in various fields, including animal production system (1). Antibiotics have been commonly used in animal agriculture to promote growth, prevent diseases, and improve feed efficiency (2). In response to the challenges of antibiotic resistance, there has been increasing interest in alternative strategies for promoting animal health and productive performance (3, 4). The use of phytobiotics, which are plant-derived substances with potential health-promoting properties (5). Plant and animal derived additives such as essential oils, plant extracts, and bioactive compounds, offers a range of medicinal benefits including antimicrobial, antioxidant, anti-inflammatory, and immunomodulatory and could be used as alternative to antibiotic and contributing to the overall sustainability of livestock industry (6–9).

Over 480 significant active chemicals have been identified as cannabinoids, the active cannabis-derived compounds with medicinal activity. Each active pharmacological ingredient in a cannabis sample has a different concentration depending on the subspecies of the plant, how the leaves were dried, when the leaves were harvested, the plant’s age, and other elements (10). The three main categories of cannabinoids are endogenous cannabinoids (endocannabinoids), herbal cannabinoids (phytocannabinoids), and synthetic cannabinoids. Cannabinoids are chemical substances that primarily act on certain cannabinoid receptors (11). Cannabidiol derived from cannabis plant has gained popularity for its potential therapeutic properties and is being explored in various industries, including agriculture and livestock sectors. The potential application of of cannabinoids in animal feed is a relatively new and expanding area of research. Some studies suggest that cannabinoids may have anti-inflammatory and stress-reducing effects, which could potentially benefit livestock.

Cannabinoid receptors are categorized into two types, cannabinoid receptor type 1 (CB1) and cannabinoid receptor type 2 (CB2), which have been associated to heterotrimeric guanine nucleotide-binding proteins (G-proteins). The effects of cannabinoids on intelligence and thinking ability, hunger, emotions, memory, perception, and motor function are correlated with the widespread distribution of CB1 receptors in the brain central nervous system (CNS). CB2 receptors are more prevalent in the immune system and peripheral nervous system than in the CNS, where they play pivotal role in the control of inflammation and pain (12). Delta-9-trans-tetrahydrocannabinol (δ9-THC), more commonly called “THC”, is the psychoactive component of cannabis that makes it a popular recreational drug. A typical cannabis plant’s component can contain up to 10% THC. One of the cannabinoid compounds known as CBD is not thought to be psychoactive and has more of a medical use (13).

Tetrahydrocannabinolic acid (THCa) and cannabidiolic acid (CBDa), present in plant during its growth, are converted to THC and CBD by heating process known as “decarboxylation” (14). Based on its cannabinoid content, cannabis is categorized into chemotype I, II, III, IV and V. High levels of the psychoactive compound 9-tetrahydrocannabinol (9-THC) are present in chemotype I, which is utilised therapeutically. Chemotype two characteristics fall between those of fibre and medicinal hemps. Chemotypes three and four are threadlike and have relatively low concentrations of psychoactive chemicals and high concentrations of nonpsychoactive cannabinoids. Chemotype V, the final group, is fibrous and devoid of cannabinoids (15). *Cannabis sativa* L. C. *sativa* L. var. *ruderalis*, var. *indica*, var. *sativa* and C. *sativa* L. are the cultivars that are currently considered as one diverse species (14).

Given that scientists are much interested in the potential health advantages of cannabinoids from *Cannabis sativa* L. in the making of food, veterinary medicine, and medicines **Table 1**. Our goal is to give a particular summary of the latest information regarding the cannabinoids and plant properties, as well as an evaluation of the cannabinoids’ potential for usage in food and medicine.

**Table 1:** Properties of cannabinoids.

NAME OF CANNABINIODS	MOLECULAR STRUCTURES	POTENTIAL HEALTH BENEFITS	ADVERSE EFFECTS	BINDING SITES	REFERENCES
Tetrahydrocannabinol (THC)	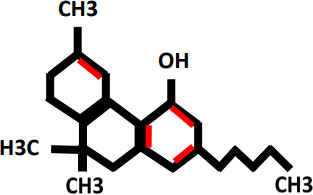	Improve sleep and stimulate appetite	Dysphoria,hallucinations,paranoia, drowsiness, uncertainty, headache, xerostomia, depression, elation, low blood pressure, Seizures	Cannabinoid receptor type 1 (CB1) Cannabinoid receptor type 2 (CB2)	(16–18)
Cannabinol (CBN)	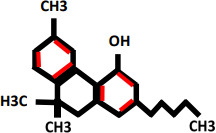	Antidepressant	Not studied yet	Cannabinoid receptor type 2 (CB2) G protein coupled receptors (GPCR)	(19)
Tetrahydrocannabivarin (THCV)	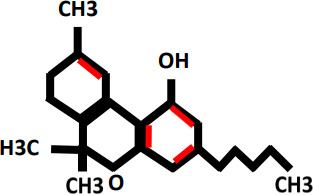	Treatment of epilepsy and obesity	vomiting, upper respiratory tract inflammations, serious mental disorders anxiety, depression and suicidal thoughts	Cannabinoid receptor type 1 (CB1)	(16, 20–22)
Cannabigerol (CBG)	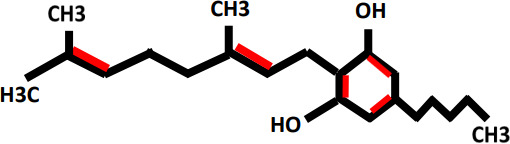	Antineoplastic	Not studied yet	Steriods like corticosterone and corticol	(23, 24)
Cannabidiol (CBD)	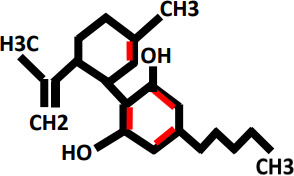	Analgesic Anti-inflammatory Anxiolytic	liver damage, somnolence, sedation, increased suicidal thoughts	Transient receptor potential vanilloid 1 (TRPV1)	(25–27)
Cannabichromen (CBC)	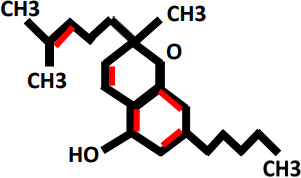	Antidepressant	Constipation inflamed intestine	Transient receptor potential vanilloid 1 (TRPV1) Cannabinoid receptor type 2 (CB2)	(28, 29)

## 2 History and origin

*Cannabis sativa* L. is among the planet’s earliest cultivars of plants. Initially utilised as a source of fodder in animal feed and as a fabric for clothing, humans eventually turned to it as a source of food and medicines (15, 30). The plant includes cannabinoids, which are bioactive substances (10). Hemp has been used medicinally in Europe since the thirteenth century. Its antiepileptic, palliative, and antiemetic qualities were discovered in 19th century (31). In terms of land use for hemp production and the quality of the items produced by the end of the 1950s, Russia and Italy were the top two countries (32, 33). Canada was among the first nations to legalise industrial hemp production, and it continues to be a major distributor and exporter of the crop, notably in the food business (33). The European Union is the world’s 2^nd^-largest cultivator of Cannabis sativa L., with centres in Romania, the Netherlands, Lithuania, and France. C. sativa L. has long been recognized as an important plant roughage resource. Hemp seeds have acquired admiration over last few years due to their high nutritional contents and presence of phytochemicals that have positive effects on human health (33).

*Cannabis sativa* L belongs to the Cannabaceae family and the Urticales order. This perennial herb is cultivated in the Boreal Hemisphere’s temperate conditions (34). Since the plant has scattered throughout the world and has been changing for generations, it is unknown where hemp first appeared to grow (15, 30, 34). There are records of *Cannabis sativa* L. cultivation and use dating back to the Neolithic era. In cave artefacts from around 700 before Christ (BCE), the first known instances of the plant’s use for therapeutic purposes were discovered. The origin of *Cannabis sativa* L. may have been in Central Asia, from which it may have migrated to the Mediterranean region, Eastern, Central Europe, especially in Afghanistan and Pakistan. According to studies, *Cannabis sativa* L. has two additional centers of species diversity; the Hindustani and European-Siberian varieties (35).

## 3 Potential of cannabinoids to address autoimmune diseases and chronic inflammation

A so-called cannabinoid system made up of certain receptors and ligands appears to exist in the immune system and brain tissues. This system mediates communication between the various tissues, along with others that use hormone and cytokine agents (36). Even though the structure and function of the cannabinoid structure have been extensively studied, there are still many unanswered questions, particularly in regards to the system’s role in immunity (i.e., immune cannabinoid system).

### 3.1 Evidences of cannabinoid receptors in autoimmune system

Cannabinoid receptors (CBRs) can be divided into at least two subtypes, CB1 and CB2. At first, pharmacological evidence implied that these receptors were present in brain tissue and was verified by cloning of CB1 using complimentary DNA from a *Ratus ratus* CNS (37, 38). Interestingly, a human immune cell line rather than the brain was used to clone the second subtype, CB2 (39). It became clear right away that the CBR system existed in immune system cells in addition to the brain cells. CBRs are grouped into 7 transmembrane G protein-coupled receptor super families (40), Moreover, recent studies suggest that they may also bind to Gs proteins, despite the fact that they convey signals via a pertussis toxin-sensitive Gi/Go inhibitory pathway (41). Notably, immune system cell signalling has been connected to G protein pathways (42). The brain and peripheral organs both have endogenous ligands for these receptors in addition to CBRs (43). Because they are structurally based on arachidonic and palmitic acids rather than cannabinoids, these molecules often have a lower affinity for CBRs than cannabinoid derivatives (44). Their existence lends credence to the current hypothesis that the entire cannabis system, which consists of endogenous receptors and ligands, regulates a wide range of physiological processes in both the brain and peripheral tissues. They are created by immunological and brain cells respectively (45).

The discovery of CB1 mRNA expression in human testis tissue provided the first evidence of CBRs being expressed outside of the brain (46). Following this, it was discovered that human peripheral blood mononuclear cells (PMBCs) and mouse solenocyte’s both expressed CB1 mRNA using reverse transcriptase polymerase chain reaction (47, 48). Additionally, it was shown that immune cells and the rat spleen expressed the second receptor subtype CB2 at higher levels than CB1 rather than the brain (39, 49). Immune system cells have different levels of CBR expression. For instance, polymorphonuclear neutrophils, B cells, CD8 cells, NK cells, monocytes, and CD4 cells are in decreasing order of CB1 expression in human peripheral blood mononuclear cell (48). Interactions between the cannabinoid systems have lately been found to follow this tendency. The expression of cannabinoid receptors and anandamide in the immune system, brain, and hypothalamic-pituitary-adrenal (HPA) axis has been demonstrated. Both receptor subtypes seem to be expressed by the immune system. Combined with other cytokines and neuroimmune hormones, the cannabis system may facilitate bidirectional communication between neural and immune tissues mouse splenocytes (50). These investigations, along with others, have contributed to the development of the current hypothesis for CBR distribution, which states that CB1 is largely found in brain and nearby structures like the pituitary (51) and peripheral nervous tissues (52), while CB2 is largely found in the immunological and reproductive systems. Along with the numerous CBR subtypes, these organs also express endogenous ligands such as anandamide. The outcome is the development of the body’s immunological cannabinoid system.

### 3.2 Cannabinoid use in auto immune diseases

Cannabinoids have been tested as a possible treatment for a number of chronic auto immune illnesses. Autoimmune deficiency syndrome (AIDS) and multiple sclerosis (MS) are two of these. The manufacturing of reliable THC chemical formulation and delivery methods that are secure to use and more potent than marijuana smoking is a main problem in the utilization of CBD in these ailments as it is known that smoking marijuana is a fundamental delivery system for THC that also transfer toxic compounds. While inhalers and cutaneous patches are already in the works, THC- and other active cannabinoids-containing medication formulations have not yet been created (53).

### 3.3 Antitumor effects of cannabimimetic agents

The patterns of hematopoietic and tumor cells development are affected by cannabimimetic substances. For instance, anandamide greatly boosts the proliferative effect of IL-3 on the myeloid cell line 32Dcl3 via a CB2-mediated mechanism (54). Anandamide, on the other hand, prevented the development of breast and prostate cancer cell lines when the levels of prolactin and nerve growth factor receptors were decreased (55). The inhibitory impact was shared by several cannabis agonists, and the CB1 receptor appeared to be implicated hence, these compounds can prevent tumor development in mice and rats (56). To demonstrate this, mice were given THC with other cannabimimetic drugs for up to 7 days following the implantation of C6 glioma cell tumors. This therapy increased survival and reduced tumor size (56). Additionally, it was demonstrated that the drug’s mode of action involves causing tumor cells to undergo apoptosis. Numerous research studies, like this one, have demonstrated that substances associated to cannabis cause apoptosis (57, 58). It’s likely that the main mode of action by cannabimimetic medicines in a variability of tissues, with malignancies, is programmed cell death.

### 3.4 Anti-inflammatory effects of cannabinoids

According to recent studies, cannabinoids and their non-psychoactive derivatives have anti-inflammatory potential in addition to their popular usage as analgesics. Oral administration of the THC-11-oic acid dimethylheptyl derivative to mice reduced both short-term and long-term inflammatory changes (59). Additionally, it has been demonstrated that this chemical has potent analgesic and anti-inflammatory properties and is well tolerated by the host when administered orally (59). In multiple studies, it has been shown that the non-psychoactive cannabinoid HU-211 reduces inflammation brought on by the release of cytokines like TNF-a (60, 61). These studies highlight a significant issue with the link between marijuana’s effects on cytokines and these chemicals’ effects on inflammation. The drug’s anti-inflammatory effects are most likely caused by a reduction in cytokine production or activity. According to Klein et al. (62), cannabimimetic drugs have a significant impact on cytokine biology and, depending on the circumstance, may have proinflammatory or anti-inflammatory effects. More study is necessary to settle these possibilities.

## 4 Role of cannabinoids to control oxidative stress in animals

**Figure 1:**
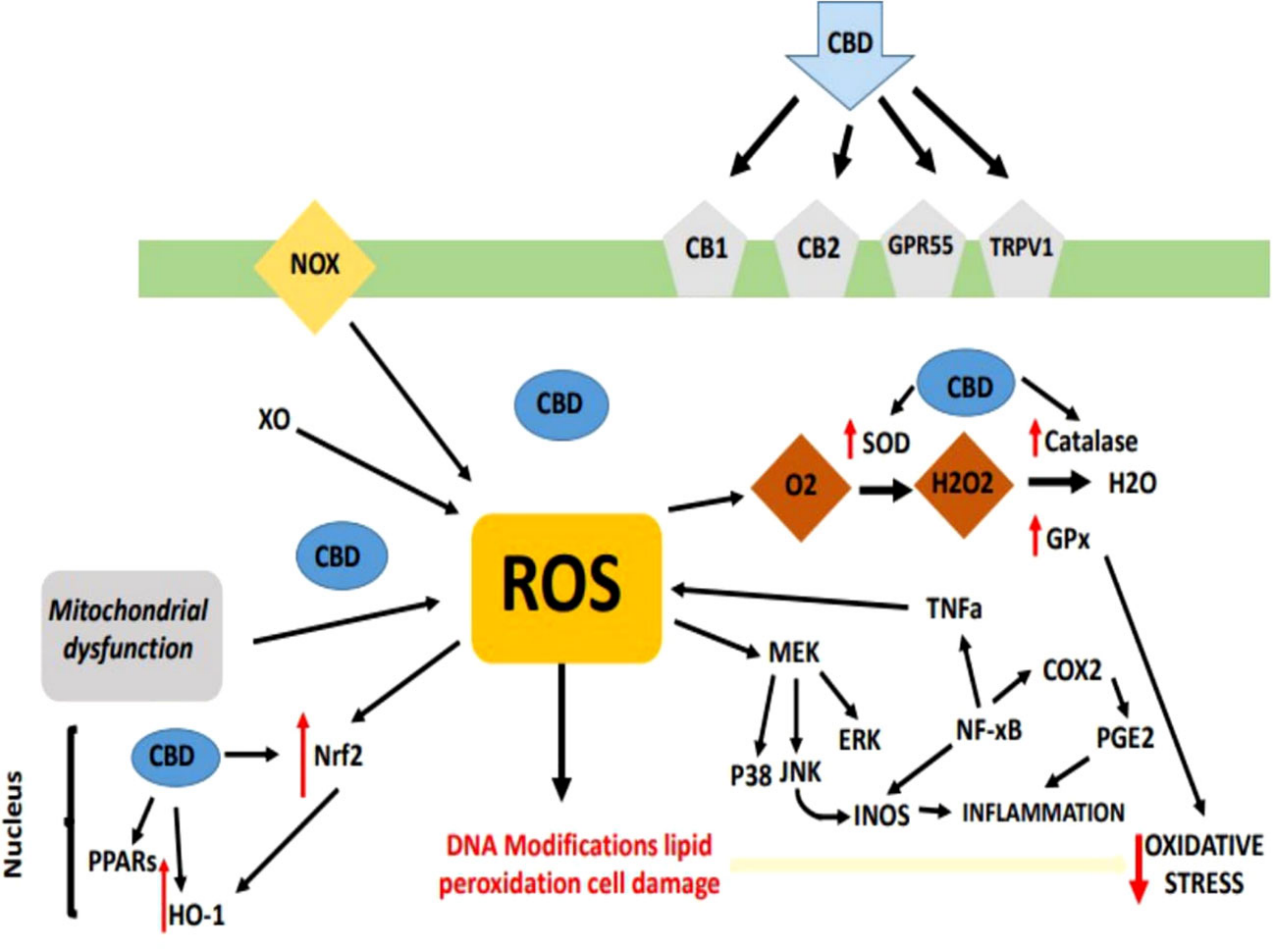
Overview of antioxidant property of cannabinoids, especially CBD, have been shown to act as scavengers of free radicals via neutralizing the reactive molecules, preventing them from causing cellular damage.

Oxidative stress as a result of emergence of free radicals have pivotal role in the causing of many ailments e.g., atherosclerosis, rheumatoid arthritis, diabetes, cardiovascular diseases, cancer, chronic inflammation, myocardial infarction, post-ischemic perfusion damage and some degenerative ailments in *Homo sapiens* (63–66). *Cannabis sativa* L. is a best resource of naturally occurring antioxidants and could be utilized in the controlling of oxidative stress. Antioxidants protect the body from the side-effects of free ions, stop the oxidation of molecules, and protects from cell damage (67–69). Now-a-days, much research has been done on hemp (*Cannabis sativa* L.), also known as industrial cannabis which is basically studied because of its chemical composition i.e. one hundred and thirty-three cannabinoids and terpenes (70). Cannabinoids such as tetrahydrocannabinol (THC), cannabinol, and cannabidiol (CBD) are potential lipophilic antioxidants (71), and their pathway of CBD and THC has been reported (72). For many years, researchers have examined and well documented the antioxidative and anti-inflammatory characteristics of cannabis in a range of tissue types and cellular models (73). The antioxidant activity of CBD is seen in **Figure 1**. Numerous studies have shown that CBD, the main non-psychoactive phyto cannabinoid in *Cannabis sativa*, has a wide range of anti-inflammatory properties and a propensity to control oxidative processes in neuropathic and inflammatory models (74).

### 4.1 Cannabinoids mode of action to control oxidative stress

CBD has both cannabinoids receptor-dependent and -independent modes of action. It also exhibits very low affinity and negligible agonist activity for both CB1 and CB2 receptors (16, 75). Peroxisome proliferator-activated receptor- (PPAR-) as a CB1/2-independent mechanism of action for CBD (76, 77), TRPV1 receptor (78), G-protein coupled receptor 55 (GPR55) (79), 5-hydroxytryptamine (5-HT) receptors (76, 80–82) and μ-/δ-opioid receptors (Kathmann et al., 2006) are discovered.

### 4.2 Evidences of CBD to control oxidative stress

**Figure 2:**
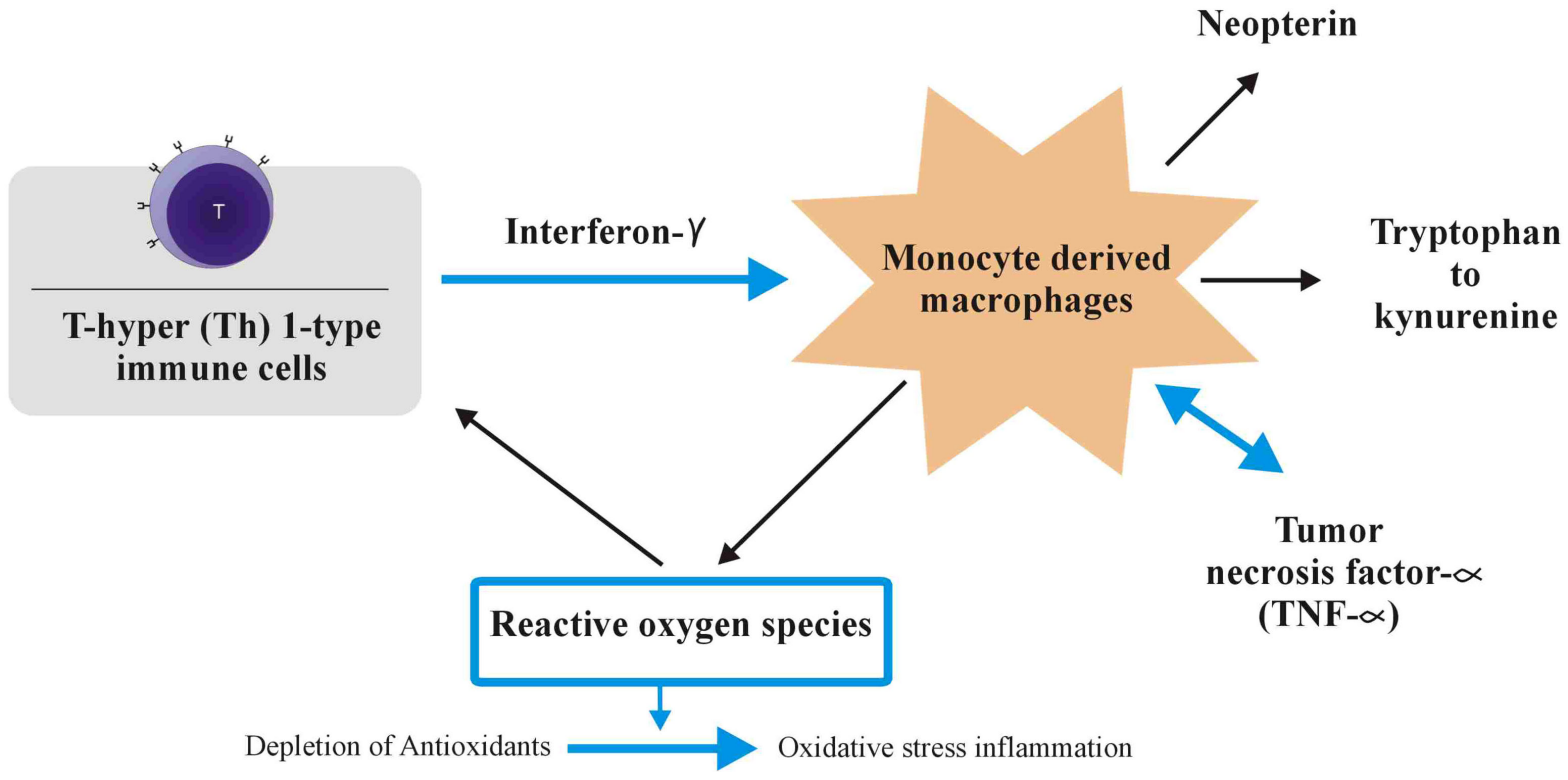
During the adaptive immune response, immune cells of the T-helper (Th)1 type that have been activated create cytokines like interleukin-2 or interferon (IFN). In monocyte-derived macrophages (M), IFN-, a pro-inflammatory cytokine, stimulates the production of reactive oxygen species (ROS), as well as the action of indoleamine-2,3-dioxygenase (IDO) and GTP-cyclohydrolase I, which are both involved in the alteration of tryptophan to kynurenine and the production of neopterin, respectively. The creation of tumour necrosis factor (TNF), which increases macrophage receptivity to pro-inflammatory IFN, is triggered by the formation of ROS, which also activate redox-sensitive signal transduction cascades. When cells’ antioxidant defences are continuously overwhelmed by ROS, oxidative stress and inflammation result.

CBD has been shown to lessen oxidative metabolism in polymorphonuclear leukocytes and nucleus pulposus cells that have been exposed to H_2_O_2_, and numerous research have suggested that CBD possesses antioxidant capabilities (83, 84), and moreover lowers pancreatic cell oxidative stress markers (85). It’s interesting to note that CBD works similarly to vitamin E (alpha-tocopheryl acetate) in reducing the generation of reactive oxygen species (ROS) in the brain after exposure to cadmium chloride (86), additionally, data suggests that it is more neuroprotective against glutamate toxicity than ascorbate and a–tocopherol (86). Due to the physiological and pharmacological variety of CBD and sign of its similar antioxidant activity to identified antioxidants, CBD is a promising medication for therapeutic immunomodulation. Following are the evidences that are collected from various researches that proves role of CBD in oxidative stress **Figure 2**.

#### 4.2.1 Role of CBD in redox equilibrium

According to a large body of research, CBD alters redox equilibrium via changing the concentration and activity of antioxidant molecules. In fact, research on CBD has demonstrated that it affects how redox-sensitive transcription factors like nuclear factor erythroid 2–related factor 2 (Nrf2) are controlled in microglia (87), keratinocytes (88) and endothelia (89), It is critical because Nrf2 is necessary for cytoprotective and antioxidant gene transcription to begin (90).

Through intrinsic methods, CBD primarily influences redox equilibrium. According to data, CBD breaks up free radical chain reactions and uses the hydroxyl groups on its phenol ring and electrophilic aromatic region to change free radicals into more innocuous molecules (91). It was demonstrated that CBD delivered electrons at a potential similar to that of well-known antioxidants and inhibited hydroperoxide-induced oxidative damage in neurons using the iron-catalyzed ROS production technique (Fenton reaction) and cyclic voltammetry (86). Using cyclic voltammetry once more, it was shown that CBD is an antioxidant on par with tocopherol and butylated hydroxytoluene, two widely used antioxidants (92). Recent evidence showing that CBD can lessen the formation of ROS by chelating the transition metal ions involved in the Fenton reaction (93). According to data, although concurrently amplifying Yo-induced ROS generation, CBD reduces the destruction of mitochondrial membrane potential brought on by anti-Yo antibodies in a way comparable to that of the ROS scavenger butylated hydroxytoluene. This shows that CBD protects against paraneoplastic cerebellar degeneration caused by anti-Yo (94). In an oxygen-glucose-deprivation/reperfusion injury paradigm, additionally, CBD has been shown to guard against energy stress on hippocampus neurons by controlling glucose uptake and triggering the pentose-phosphate pathway (95).

#### 4.2.2 Role of CBD in controlling protein expression

Recent research has demonstrated that CBD can target the expression of Kelch-like ECH-associated protein 1 (Keap1) and Nrf2 in pulmonary artery smooth muscle cells, potentially boosting its antioxidant benefits in a model of pulmonary arterial hypertension (96). Furthermore, CBD regulates the expression of the induced antioxidant enzyme heme oxygenase-1 (HO-1) in keratinocytes (97), adipose tissue-derived mesenchymal stem cells (98), neuroblastoma cells (99) and smooth muscle (100). This may have an impact on how effectively this Phyto cannabinoid regulates the level of ROS in cells. In fact, irrespective of CB receptors, in a time- and concentration-dependent approach, CBD dramatically upregulates HO-1 mRNA and protein expression in human umbilical artery smooth muscle cells (89).

#### 4.2.3 Role of CBD in activity of superoxide dismutase

Previous studies have shown that CBD can control the activity of the superoxide dismutase (SOD) enzyme as well as the Cu, Zn, and Mn-SOD enzymes (88, 101). CBD’s vasorelaxant effects are diminished by a Superoxide Dismutase (SOD) inhibitor, demonstrating that SOD increases CBD’s vascular activities (102). Additionally, by raising glutathione (GSH) levels and concurrently raising GPx and SOD1 activity after injury, CBD reduces hippocampus oxidative damage during oxygen-glucose deprivation/reperfusion injury (95). *In vivo* injection of CBD mitigates the decline in the oxidized glutathione ratio (GSH/GSSG) in diabetic mice’s cardiac tissue (101). Further shields against GSH depletion in cardiac tissue after doxorubicin cardiotoxicity (103).

#### 4.2.4 Role of CBD in activity of ROS

Data shows that CBD has an inherent capacity to scavenge free radicals. In fact, it has been demonstrated that CBD reduces LPS’s ability to cause ROS in microglia (104). Additionally, CBD inhibits the production of mitochondrial superoxide in human coronary endothelial cells stimulated by high glucose levels and lowers the production of mitochondrial ROS after hippocampal oxidative injury caused by oxygen-glucose deprivation/reperfusion injury (105). In models of retinal neurotoxicity, CBD has been shown to have neuroprotective benefits by directly reducing N-methyl-D-aspartate (NMDA) mediated oxidative stress and maybe by targeting the synthesis of nitro tyrosine, a byproduct of tyrosine nitration (106). Similarly, it has been demonstrated that CBD has ROS scavenging properties created by H_2_O_2_-driven ROS in keratinocytes and oligodendrocyte progenitor cells, shielding them from H_2_O_2_-induced cell death (107, 108). Recently, it was demonstrated that CBD had a comparable impact on H_2_O_2_-induced ROS in intestinal cell monolayers (109). Furthermore, findings show that CBD works similarly to -tocopheryl acetate in reducing brain ROS generation after exposure to cadmium chloride (110). Moreover, CBD dose-dependently lessens the generation of ROS in neurons caused by β-amyloid (111). Parallel to this, CBD has been demonstrated to lessen cisplatin’s induction of renal nitro tyrosine synthesis in a model of nephrotoxicity (112). It has additionally been demonstrated to dose-dependently decrease the ROS generation brought on by tert-butyl hydroperoxide in keratinocytes (97). Accordingly, polymorphonuclear leukocytes exposed to chemotactic peptides produce less ROS when CBD is present (83) additionally, administering CBD *in vivo* reduces the level of lipid peroxides and ROS in diabetic mice’s cardiac tissue (113). Last but not least, new study by Baeeri and colleagues (85) demonstrates that CBD can serve as a free radical scavenger in response to a range of stressors by decreasing age-related increases in ROS production in pancreatic islets.

## 5 Nutraceutical effects of cannabinoids

Hempseeds and seed meal derived from Cannabis sativa have proven to be significant contributors to the Old World’s food resources. These seeds are abundant in essential fatty acids, such as omega-3 and omega-6, making them a nutritious source of dietary oil. Moreover, they offer a substantial amount of protein and fiber, enhancing the overall balance on nutrients and bioactive compounds. The prospective usage and advancement of *Cannabis sativa* seed as a source of nutrition for human and house animals was halted after the forbidding of Cannabis variants growth in the late 1930s (114).

Whole hempseed typically contains 20 to 25 percent protein, 25 to 35 percent carbohydrates, along with 10 to 15 percent insoluble fibre, and 25 to 35 percent oil (taken via cold pressing the seeds or by extraction of oil) (114, 115). Regarding the nutraceutical abilities of cannabis by-products, various outcomes of their inclusion to basal feed have been hypothesized, includes a decrease in the occurrence of tibia deformation in egg-laying chicks and hens, an enhanced serum lipid profile, a protective impact against the onset of hepatic disease, an anti-microbial activity, an improvement in anti-oxidative systemic condition, and an anti-inflammatory action (116–120). However, additional work and study is required to establish all of these beneficial effects.

## 6 Effect of cannabinoids on nutrient digestibility, feed efficiency and live weight gain

The overall digestibility of feed or distinct nutrients is precisely known as the amount or percentage that is not eliminated in fecal waste hence considered to be retained by the organism. There were no negative impacts on digestibility due to the existence of any secondary compounds in *Cannabis sativa* straw (121). However, it is unclear why the digestibility of dry matter (DM) and organic matter (OM) has improved. In comparison to the hemp-containing pellets, the control diet exhibited elevated concentration of polyphenolic chemicals. Polyphenolic compounds such as tannins diminish the digestibility of food by binding to gastric enzymes and dietary proteins, as compared to a controlled diet (122). Hemp contains flavonoids, which can lower DM digestibility (123, 124). Digestibility and lignin contents are inversely associated (125, 126). When hemp stubble was added to the pelleted diets, the lignin content increased, but there was no negative correlation between digestibility and lignin level. The digestibility of a diet is also impacted by variations in the neutral detergent fiber (NDF) and digestibility of the forage products. Oat straw typically has an NDF digestibility of above 20 percent (127), in contrast to *Cannabis sativa* stem which is 12.7 percent (121), suggesting that oat straw could be more easily digested than hemp stalk. More research into the digestibility of *Cannabis sativa* straw is necessary to understand the changes in apparent DM, OM, NDF, and Acid Detergent Fiber (ADF) digestibility’s as a result of *Cannabis sativa* straw addition in the pelleted diets. In order to study, cannabinoid role in controlling feed conversion ratio, cold-pressed *Cannabis sativa* seed cake was studied as a protein feed for young cows and finishing steers. Effects on feed intake, live weight gain (LWG), faecal traits and carcass traits (steers only) were investigated. Animals fed Cannabis sativa seed cake consumed more NDF than those fed Glycine max diet (*P* < 0.05). Lower feed efficiency as a result of higher feed intakes and equivalent LWG was observed in calves given *Cannabis sativa* (*P* < 0.05). In summary, developing cattle who are aggressively fed *Cannabis sativa* seed cake instead of *Glycine max* meal produce equivalent amounts of milk and have better rumen functions (128).

## 7 Role of cannabinoids in nutrient absorption, metabolism, and excretion

**Figure 3:**
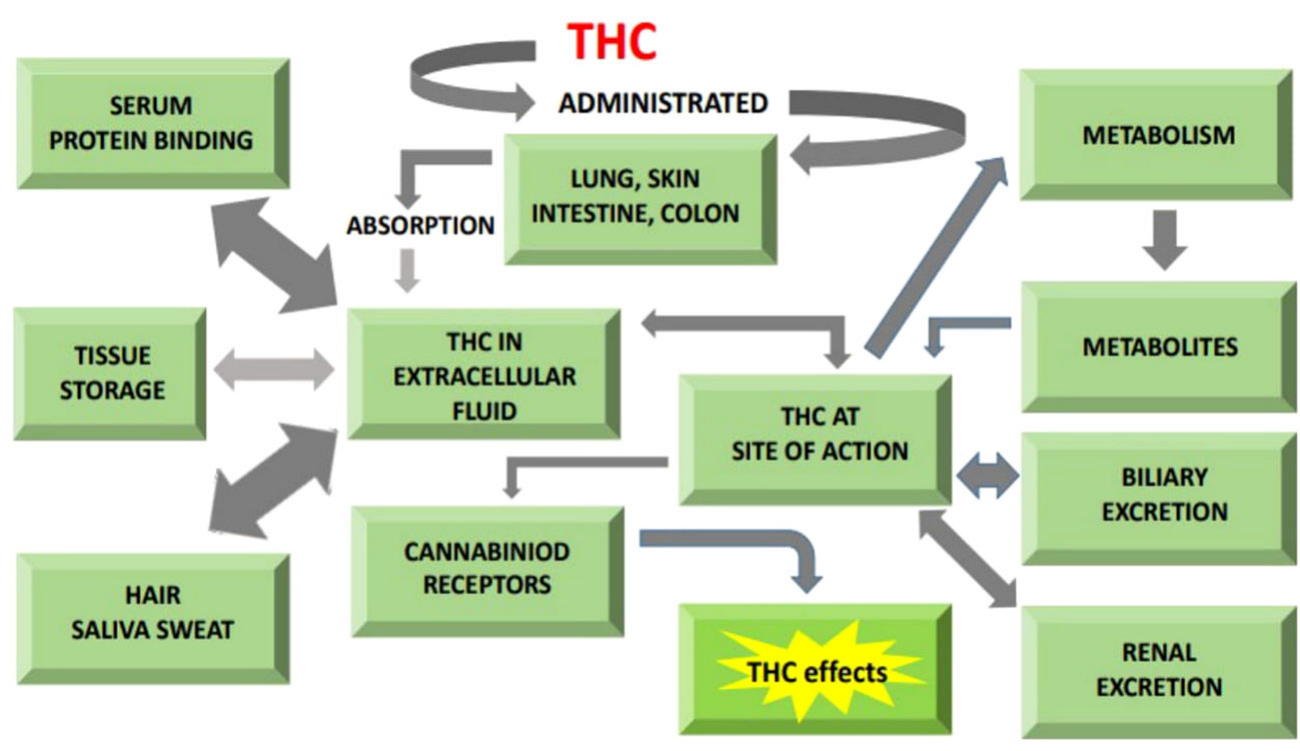
General illustration of pathway showing cannabinoids role in absorption, metabolism and excretion.

The ECS clearly plays a critical effect in macronutrient metabolism, hence regulating feed consumption and body energy homeostasis (129, 130). General pathway illustrating role of THC in energy metabolism is given in **Figure 3**.

### 7.1 CB1 activation stimulates appetite and nutrient uptake

CB1 promotes intake in animals by causing orexigenic peptides to bind to hypothalamic neurons and preventing the addition of anorexigenic proteins (131). After feeding, the adipose tissues (AT) releases leptin hormone in this metabolic process, which binds to the hypothalamus and causes the release of anorexigenic peptides (132). According to studies, leptin resistance and hyperleptinemia in a diet-induced obese mouse model were reversed by peripherally-restricted CB1 inverse agonist (133). These findings show how CB1 can inhibit the hypothalamic leptin sensitivity and satiation signaling pathways thus playing pivotal role in nutrient uptake (134).

The gastrointestinal tract contains all of the components of the ECS. When food is first taken into the mouth during a meal, cephalic-phase reactions happen to anticipate and prepare for optimal digestion. The orexigenic hormone ghrelin, which is released when the gastric CB1R is activated, raises the perception of fat and encourages consumption of fat (135). Furthermore, in both rodents and humans, the ECS in the gut may change cholinergic transmission to the colon, lowering intestinal motility (136). Additionally, the CB1Rs’ anti-inflammatory properties make the ECS a possible enhancer of food absorption in the GI tract (136).

### 7.2 NAPE-PLD, the intestinal barrier, and nutrient absorption

Nutrient absorption in rumens is increased by improving gut epithelial barrier and microbial function are affected by adipose tissue levels of N-acetylphosphatidylethanolamine phospholipase D (AT NAPE-PLD), which in consideration enhances energy storage function in a periodic way (137). The intestinal epithelium has a pivotal role in the absorption of nutrients, hormone release, and synthesis of endocannabinoids (eCBs), all of which affect metabolic activity (138). Few minutes’ nutritive fatty acids (FA) exposure in the stomach in monogastrics causes jejunal anandamide (AEA) mobilization and FA transport into the duodenum, which enhances oleoylethanolamide (OEA) production (139). Endocannabinoid system (ECS) activation in the stomach enhances adipogenesis in addition to enriching eCB production (140). The intestinal ECS lowers LPS transferring, barrier breakdown, gut inflammation, and dysbacteriosis of gut microorganisms in monogastric animals (140).

When released from the rumen epithelium, lipopolysaccharide (LPS) in dairy cows crosses the intestinal barrier and enters the bloodstream. Elevated levels of endotoxin in the bloodstream lead to substantial changes in metabolism and provoke systemic inflammation (141). The same study found that circulating LPS levels related to blood glucose and non-esterified FA levels (141), and these gains are followed by dairy cows consuming less dry matter (142). It is interesting to note that local CB1 activation reduces the amount of LPS that enters the body, which may increase appetite and reduce inflammation in milking cows.

## 8 Role of cannabinoids in lipid metabolism

One of the most major health issues in Western countries is obesity, and the discovery that the endocannabinoid (EC) system is involved in the control of energy balance and the focalization of fatness is a huge improvement in our knowledge of this issue. Ancient medicine was aware of the impact of plant-extracted CBD on individual weight or body mass and appetite, but it wasn’t until recently that the mechanisms underlying these effects were understood. This was made feasible by the exact EC receptors’ identification as well as the endogenous ligands anandamide and 2-arachidonoylglycerol (2AG) (38, 143–145). Numerous experimental studies have shown that ECs are present in adipose tissue and other membrane tissue involved in the energy metabolism. This information provides another hint to understanding adipose tissue function in *Homo sapiens* obesity (146, 147).

Multiple evidences suggest that endogenous cannabinoids are appropriate for the membranous control of lipid management in fat tissue, which follows the revelation that these molecules are taking part in hypothalamus regulation of food intake (147–149). Rimonabant, a sepecific CB1 blocker, has been the subject of numerous phase-III clinical trials, all of which have demonstrated that inhibiting CB1 lowers body mass in fat specimens and improves cardiovascular risk elements in obese and diabetic patients (150–153). A sufficient amount of both fatty acids and glucose must reach fat cells in order to store and expand triglycerides. To feed lipid substrates to fat cells, fatty acid flux from chylomicrons and very-low density lipoprotein is mediated by lipoprotein lipase (LPL). The crucial processes of creating the glycolytic intermediate a-glycerophosphate required for triglyceride synthesis are insulin-dependent glucose transporter (GLUT4) translocation and glucose transport. In both of these pathways, insulin is in charge. Enough insulin sensitivity and the activation of its downstream machinery are thus necessary to permit adequate fuel channeling to fat cells (154).

CB1 receptor is not found on preadipocytes however, upon differentiation, adipocytes rapidly exhibit its expression. This has been seen in both primary *Homo sapiens* adipose cell and primary cells and cell lines from rodents (155). It is debatable whether adult adipocytes express CB2. While some scientists discovered considerable expression of CB2 in differentiated adipocytes, others were unable to (146). It is plausible that predispose cells, invasive macrophages, or vascular cells are the source of CB2 mRNA in fat tissue extracts because CB2 is expressed at modest levels in fat tissue biopsies as well (146). Adipose tissue and fat cells both express CB receptors as well as the enzymatic machinery needed to create and break down endogenous cannabinoids locally (156, 157). In primary mouse adipocytes, activation of CB1 increases lipoprotein lipase activity (158). As a result, there would be a greater inflow of free fatty acids into adipocytes for the synthesis of triglycerides. They found that the strong CB1 agonist HU210 stimulates the creation of intracellular lipid droplets in 3T3-F442A cells, demonstrating the importance of CB1 and ECs in the growth of neutral lipids in fat cells (156). Adipocytes produce more 2AG and anandamide before adipose cell differentiation occurs, supporting the idea that this system is responsible for causing preadipocytes to convert to adipose tissue (156).

The entrance of glucose into fat cells is also encouraged by CB1 activation. CB1 activation increases glucose absorption in human primary adipose cells, and this action is achieved by Glucose transporter type 4 (GLUT4) moving from an intracellular compartment to the plasma membrane, which is where it is located. Additionally, the cannabinoid-stimulated glucose uptake in fat cells is mediated by the same molecular mechanism as drives insulin-induced glucose uptake stimulation of PI3-kinase. Actually, the benefits of CB1 activation on glucose absorption are totally negated by the inhibition of this enzyme by wortmannin. Additionally, the absorption glucose into the fat cells is mediated by an increase in intracellular calcium from the surrounding environment (146). In studies conducted in calcium (Ca) free medium or with the Ca chelating reagent ethylene glycol tetra-acetic acid (EGTA), The translocation of GLUT4 and the absorption of glucose were unaffected by CB1 activation. Rimonabant fully offset the effects of the CB agonist on glucose absorption. The extent of the ECs’ impact on glucose absorption was between 40 and 50 percent that of insulin. However, it is uncertain what physiological consequences EC-induced glucose clearance by fat cells would have. Although the EC’s effect as insulin in fat cells is expected to be important for triglyceride accumulation and preadipocyte formation, the influence on the body’s ability to handle glucose should be minimal. Rimonabant-based *in vivo* investigations have repeatedly demonstrated that provoking CB1 did not degrade insulin resistance in obese people, instead causing weight loss and a reduction in the size of fat tissues and perhaps adipose fat cells increased whole-body insulin sensitivity (159).

Mice lacking CB1 receptors (CB1/) are thin and unaffected by high-fat diet (160). Similar to how rimonabant, a selective CB1 receptor antagonist, causes reductions in body weight of obese rats over time, however after a brief initial 1–2-week weight loss, food intake returns to normal (160, 161), indicating that the stimulation of energy metabolism by CB1 receptor blockage results in a reduction in fat content. If we take a holistic view of the body, this situation may result from higher energy expenditure along with enhanced oxidative capability of many tissues, in specific the brown adipose tissue, the liver and skeletal muscle. This might be explained by at least three causes if just white adipose tissue is taken into account: The first three alterations are an increase in lipolysis, a decrease in liposynthesis, and an increase in fatty acid oxidation inside the fat cell. Several pieces of evidence show that the CB1 blockage increases lipolysis *in vivo*. A single-dose study on postprandial rats revealed an instant rise in free fatty acids (FFAs), conclusively demonstrating an underlying pharmacological impact of rimonabant to induce lipolysis instead of a secondary one brought on by a decline in intake and after-starvation post-absorptive metabolic alterations in intermediate metabolism (162).

In conclusion, several evidence unequivocally demonstrate that EC and CB1 receptor levels increase during adipocyte differentiation (156, 163, 164). CB1 activation causes pre-adipocytes to differentiate more quickly (156). We propose that elevated lipogenesis is a result of the EC system’s overactivity stimulating Lipoprotein lipase (LPL) activity (158), an improvement in insulin sensitivity, as well as a faster rate of glucose absorption and utilization (146, 164) and a fatty acid synthase activation (147). The AMP-activated protein kinase (AMPK) and eNOS-dependent mitochondrial biogenesis in adipose tissue are also inhibited by this overactivation, which reduces ATP generation and oxidative metabolism of energy sources. This process may be reversed by blocking the adipose CB1 receptor, which would reduce adiposity and weight growth. This would provide rimonabant with a fresh, as-yet-unknown mode of action for reducing body weight (165).

## 9 Potential of cannabinoids to modulate rumen microbiome to enhance expression of fibrolytic genes

The phrase “microbiome” refers to the collective genome of microbial communities, or “microbiota,” which are connected to people, animals, and plants. The influence of microbial communities in determining the host immune system and fitness has come to light in recent years (166). There are similarities between the control of host gene expression by the gut and root microbiota (167, 168), catabolic genes that increase their hosts metabolic capabilities (169, 170), and the control of dangerous pathogens (171).

Ruminants account for a sizable portion of all domesticated animal species in the world and the fundamental producers of milk, meat and other by products. Ruminants are able to digest enormous number of plant polysaccharides because of the variety of bacteria that can be found in the rumen. The rumen, which is home to a range of microorganisms like as bacteria, archaea, fungi, viruses, and protozoa, has evolved into a prolific fermentation vessel for the breakdown of cellulose (172, 173), they interrelate and importantly affect ruminants health. Around 95% of all rumen microorganisms are bacteria, which rule over the diverse domains of the rumen’s microbiome (174). Microbes play a key role in the rumen fermentation process, which changes the content and quality of milk and meat as well as the productivity of the animal (175).

To break down the intricate plant polysaccharides, rumen microorganisms create a variety of fibrolytic enzymes known as Carbohydrate-Active Enzymes (CAZymes), which include exoglycanases, glucosidases, endoglucanases, and hemicelluloses. Technologies for high throughput sequencing (HTS) are widely utilized to tackle the complex procedure of lignocellulose breakdown in ruminants. With a greater knowledge of the rumen microbial population, In the cattle industry, issues with ruminant nutrition and environmental issues may be tackled. Number of metagenomics investigations have documented different types of fibrolytic enzymes found in the rumen of yak’s, reindeer, Jersey cow, Angus cattle, and buffalo (173, 176–178). In-depth scholarly studies on metagenomic analysis on CAZymes profile in rumen of Holstein-Friesian crossbred cattle feeding with just finger millet straw are not yet available, though.

The rumen is a special natural environment due to the genetic diversity of fibrolytic enzymes from microbial origin that break down plant polysaccharides. An investigation was conducted to determine the main cell wall-degrading enzymes in plants and the associated rumen microbiomes taxonomic profiles (179). Through a comprehensive metagenomics sequencing method, the rumen microbiota of cattle and the carbohydrate-active enzymes were divided into functional groups. The candidate genes encoding fibrolytic enzymes from various classes of carbohydrate-binding modules, glycoside hydrolases, polysaccharide lyases, carbohydrate esterases, glycosyltransferases and auxiliary activities were found through analysis of the assembled sequences using the carbohydrate-active enzyme analysis toolkit. A large fraction of the CAZymes were produced by bacteria from the genera *Prevotella, Fibrobacter, Bacteroides, Clostridium*, and *Ruminococcus*, according to phylogenetic analysis of the contigs that encode the CAZymes (179). The findings showed that the CAZymes and the rumen microbiome of cattle are extremely complex, structurally related, but different from those of other ruminants in terms of content. The rumen microbiota’s distinctive traits and the enzymes produced by the residing microorganisms provide chances to increase ruminants’ feed conversion efficiency and function as a repository for crucial industrial enzymes for the synthesis of cellulosic ethanol (179).

## 10 Potential of cannabinoids as a feed additive to enhance animal performance

The European Food Safety Authority (EFSA) panel on Additives and Products or Substances used in Animal Feed stated in its scientific opinion that hempseed and hempseed cakes might be used in animals feed, though there may be differences in rate of incorporation in diet depending on the specie (180). Animal feed may be supplemented with hemp oil, a rich source of vital fatty acids, meanwhile seeds and hempseed cakes can serve as protein and fat sources. The hemp plant produces cannabinoids, terpenophenolic compounds that are closely related to the pharmacological effects of cannabis (181). The bract covering the seed is where hemp has the most THC and other cannabinoids (182). Cannabinoids may be present in hemp seed products in substantial amounts if the hemp seed varieties are not carefully chosen, grown, processed, and handled. For instance, during cold pressing, cannabinoids can be absorbed by hemp seed oil. Cannabinoids from the resin of the flowers or leaves can also be transferred to the seeds during processing and handling.

*Cannabis sativa*, with the exception of the seeds and roots, produces cannabinoids in glandular organs (trichomes) that are dispersed across the whole surface of the plant. Trichomes are heavily concentrated in the area of influorescence, in the veins of the leaves, and on the sides of the leaves. They contain essential oils, highly polymeric phenols, terpenes, waxes, and resin that contains 80 to 90% cannabinoids. Delta-9-tetrahydrocannabinol (THC), the primary psychoactive substance, is primarily present in the inactive precursor form delta-9-tetrahydrocannabinol acid (THC-A), which may account for up to 90% of all cannabinoids in hemp plants produced in Europe (183). Cannabinol (CBN) and cannabidiol (CBD) are the other two key active ingredients among the 60 additional cannabinoids that have been found. *Cannabis sativa* phenotypes can be identified by their THC + CBN/CBD ratio. The ratio of hemp types grown for fibre production is less than 1, whereas variants grown for cannabinoids show a ratio greater than 1 (184). The plant’s cannabinoid content fluctuates according on its vegetative state of development, cultivation conditions (temperature, humidity), and other factors.

When the hemp leaf is used as forages (for cattle, for example), whether in whole or in part, the animals may be exposed to THC at levels higher than those resulting from consumption of the top portion of the same variety classified and assessed for control under the same regulation. In terms of hemp seeds, it has been demonstrated (185), that the majority of THC was discovered on the outside of the seeds due to contamination with plant debris, probably as a result of physical contact with the plant leaves during processing. Numerous research has examined the effects of consuming hempseed or its derivatives on farm animals, albeit the outcomes were not every time obvious. Here is a summary of the most telling research, broken down per animal species.

## 11 Protection against endotoxins and lipopolysaccharide’s challenge

Immune challenges include several pathophysiological situations including stress, endotoxemia, and inflammatory illnesses, which affect how neuroendocrine factors are produced and released normally (186). Lipopolysaccharide (LPS), a gram-negative bacterial endotoxin is an effective inducer of catecholamines, prostaglandin and proinflammatory cytokines to be released (187). It is therefore widely employed to elicit immunological challenge, which in turn affects neuroendocrine systems. During systemic infections, proinflammatory cytokines react to peripheral signals and cross the blood-brain block or fenestrated capillaries in specific areas of the brain to enter the central nervous system (188, 189). Additionally, the brain produces cytokines in the presence of other cells, primarily astrocytes and microglia, but also neurons and endothelial cells (190).

The primary center that receives a multitude of peripheral signals is the hypothalamus because it is the area of the brain where the majority of neuroendocrine factors that control essential pathophysiological activities are produced. In actuality, infectious organisms, antigens, and the LPS challenge quickly engage the immune system, causing it to produce interferon gamma and cytokines that are subsequently transported into the brain where they influence the function of the hypothalamus (191). It is widely known that the release of corticosterone from the hypothalamic-pituitary-adrenal axis, which is activated by the immunological response, regulates the cardiovascular, metabolic, neuronal, and immune systems (192, 193). Last but not least, glucocorticoids create a negative feedback loop that controls both their own production and the immune system (194, 195). It’s important to know that endocannabinoid signaling appears to be tightly linked to the proper operation of the hypothalamic-pituitary-adrenal axis (196–199).

The role of the ECS in innate reactions in case of inflammation and brain functions is particularly intriguing (200, 201). Numerous studies have demonstrated that various organs and tissues produce endocannabinoids as a result of infection and inflammation (202), which act as moderators to control the activated neuroimmune response (203). The profiles of endocannabinoids change significantly during a variety of pathological circumstances, such as Parkinson’s and Alzheimer’s disease, amyotrophic lateral sclerosis (ALS), multiple sclerosis (MS), traumatic injury, stroke, and bacterial and viral infections of the central nervous system due to the inflammation-modulating and the way these substances work to reduce pain (204). Additionally, endocannabinoids influence neuroendocrine function. Endocannabinoids must be produced “on-demand” for neurotransmission to be fine-tuned under both resting settings and immunological challenges that regulate the release of neuropeptides, neurotransmitters, and hormones. The formation of ECS differs based on the desired response in both circumstances, and the ECS functions as a intermediary for the transmission in between glial cells and neurons to produce the most feasible neuroendocrine responses in every scenario respectively (205).

## 12 Effects on poultry health and performance

### 12.1 Broilers

After C. sativa seeds were added to a basal diet at rates of 10 and 20 percent, broilers’ body weight dramatically increased when related to animals fed with the basal feed alone. In contrast to the control group, animals fed a diet containing hempseed had a lower feed intake and a higher feed conversion rate. The higher hempseed content resulted in the best growth performance. In contrast, the broilers body mass was considerably lower than in the control group at hempseed concentrations lower than 5 percent (206). Mahmoudi et al. (116), also observed a decrease in average daily intake and growth in broilers given 2.5 percent hempseeds over the first twenty-one days of treatment, but no change was noted in weight gain with diets at 4 and 7.5 percent (117). Neither hemp oil up to 6 percent nor *Cannabis sativa* seed cakes at 10 percent and 20 percent improved the development performance of hens in the experiments (207, 208). Effects of adding 5 and 15 percent *Cannabis sativa* seed cakes to broiler diets were investigated. Comparing the greater dose to diets without *Cannabis sativa* seed cakes, the researchers discovered a detrimental effect on broiler development but no variations in carcass weight or the ratio of breast to thigh meat (209).

### 12.2 Layers

The majority of authors came to the conclusion that adding hemp products to chicken diets had no detrimental effects on the bird’s performance. Several research has also looked into how adding hemp to eggs affected their levels of saturated fatty acid (SFA) and monounsaturated fatty acids (MUFA), polyunsaturated fatty acids (PUFA) and essential fatty acids (EFAs). The concentrations of linoleic acid (LA) and α-linolenic acid (ALA) increased linearly with the addition of 5, 10, or 15 percent *Cannabis sativa* seed cakes to the diet (210), while SFA and MUFA levels decreased. Neijat et al., 2016 examined the addition of *Cannabis sativa* seeds (ten, twenty and thirty percent) and *Cannabis sativa* seed oil (4.5 and 9.0%) and discovered that the highest amounts of *Cannabis sativa* seeds and *Cannabis sativa* seed oil significantly increased the amount of ALA and docosahexaenoic acid (DHA) in egg yolks when compared to a control group.

The fatty acid profile of egg yolks changed in the study by depending on whether *Cannabis sativa* oil or *Cannabis sativa* seed cakes were included in the diet: While ALA was greater than the control and lower than the Cannabis sativa seed group, LA was higher with Cannabis sativa seed cakes than with Cannabis sativa seeds and the control group. Oleic acid levels in the yolk were lower and MUFA concentrations were lower when chickens were given both hemp derivatives (211). The same study also found that eggs from laying hens fed a diet enriched with hempseeds or hempseed cakes contained higher levels of -tocopherol, indicating a higher antioxidant potential (211). Last but not least, it was discovered that including 25 percent *Cannabis sativa* seed in the diet of hens enhanced the -6/-3 ratio in egg yolks. Up to 12% of laying hen diets could contain hemp oil without having a negative impact on performance metrics or the flavor and aroma characteristics of cooked eggs (208, 212).

## 13 Effects on health and performance of ruminants

The effects of include hempseed cakes at variable amounts (143, 233, and 318 g/kg dry matter) in the diets of dairy cows were evaluated. When the cows received an increment of 143 g/kg in comparison to the control group animals, who were given hempseed oil, their milk production rose (213). The rate of dietary crude protein conversion into milk protein declined as hempseed cake consumption increased, which prompted the authors to draw the conclusion that adding 233 or 318 g/kg had no positive effects on milk performance (214). In contrast to cattle fed “normal diets,” other studies found no differences in weight gain when whole hempseeds or hempseed cakes were included to the diet (213, 215). However, hempseed meal might be regarded as a superior naturally occurring rumen crude protein (216). All things considered, findings suggest that hempseed cakes have better rumen performance than control diets, perhaps as a result of their higher fiber content and lower starch content (215). Studies have shown that including hempseed oil in a hay-based dairy goat diet at a rate of 4.70% increased the milk fat content, while increasing conjugated FA and PUFA proportions, but it had no effect on milk yield (217).

## 14 Potential of cannabinoids to modulate metabolic signaling pathway

In the brain, 2-Arachidonoylglycerol (2-AG) is present at a baseline level that is roughly 1000 times greater than AEA. Altering the metabolism of 2-AG, but not AEA, through pharmaceutical means has a notable impact on endocannabinoid-mediated retrograde signaling. These data lead to the hypothesis that the central nervous system’s (CNS) have naturally occurring ligand for cannabinoid receptors (CBRs) is 2-AG (218–220). AEA, however, has been demonstrated to independently activate transient receptor potential vanilloid 1 (TRPV1), inhibit l-type Ca2+ channels, and negatively regulate 2-AG production and physiological consequences in the striatum, highlighting its critical function in the control of synaptic transmission (221).

Depolarization-induced suppression of inhibition (DSI)/excitation (DSE) was the first conclusive evidence for retrograde endocannabinoid signaling (222). Furthermore, it was demonstrated that both short-term depression (STD) and long-term depression (LTD) include activation of endocannabinoid system in excitatory and inhibitory synapses (223, 224). In most situations, increasing intracellular Ca2+ concentrations and active Gq/11-coupled receptors trigger the synthesis of 2-AG, which then initiates endocannabinoid-mediated retrograde signaling (224). Then, by a procedure that is not completely understood yet, before reaching the presynaptic terminal and interacting with the CB1R, 2-AG is entered into the extracellular space and travels through it. Activated cannabinoid receptor 1 (CB1R) reduces neurotransmitter release by inhibiting voltage-gated Ca2+ channels, which reduce presynaptic Ca2+ influx, and adenylyl cyclase (AC) and the subsequent cAMP/PKA cascade, which is implicated in LTD (222–224). 2-AG must be degraded by monoacylglycerol lipase (MAGL), which inhibits signaling by being expressed in certain synaptic terminals and glial cells (223–225).

**Figure 4:**
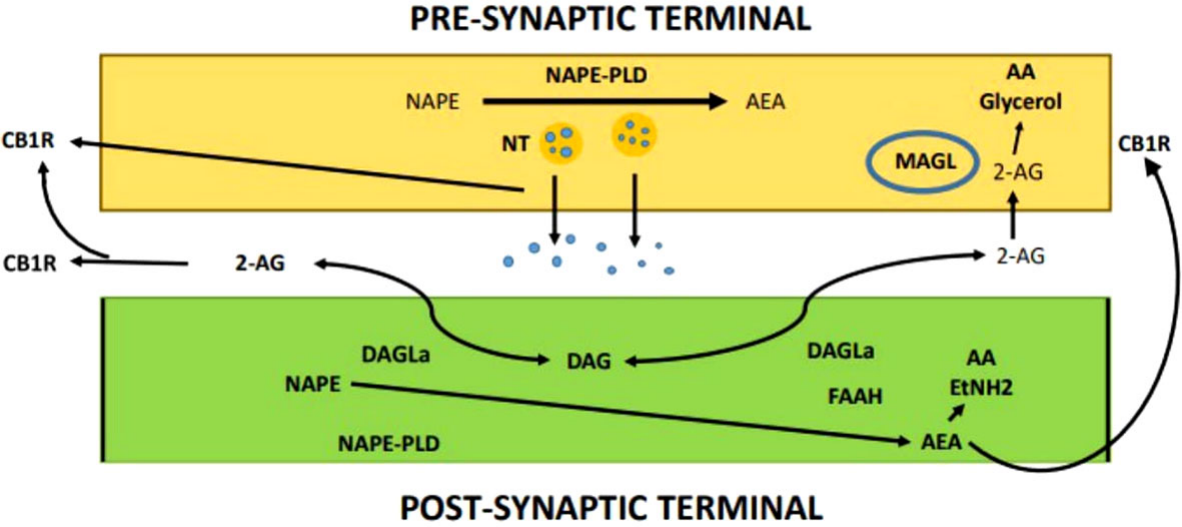
Endocannabinoid-mediated synaptic transmission.

It has been demonstrated that AEA plays a variety of roles in endocannabinoid-mediated synaptic transmission (**Figure 4**). TRPV1 is a complete agonist of AEA, and it is thought to play a role in endocannabinoid signaling (218). The effect of AEA’s negative regulation of 2-AG metabolism can be mirrored by TRPV1 activation (226). A tonic function for AEA as an endocannabinoid is also supported by the fact that chronic fatty acid amide hydrolase (FAAH) blocking causes persistent agonist of the endocannabinoid system without lowering CB1R appearance, which is the opposite of MAGL antagonism (227). Independent of the type of synaptic transmission or the length of the transmission, endocannabinoids play a significant role in suppressing synaptic transmission through a variety of methods (223, 224). A subset of neocortical interneurons, pyramidal neurons, and hippocampal cornu ammonis (CA1) neurons, as well as CB1R-dependent self-inhibition in postsynaptic neurons, have all been identified (228–230). The ability of microglial cells and astrocytes to make 2-AG or AEA has been demonstrated in earlier research, it is currently unknown, nevertheless, whether these endocannabinoids are involved in the control of synaptic transmission (231). However, despite studies demonstrating the existence of cannabinoid type 2 (CB2R) in the brain, it is still largely unclear how CB2R contributes to endocannabinoid-mediated synaptic transmission (232–234).

The CB1R modifies the working of various ion channel types (235, 236). In cultured *Ratus ratus* primary hippocampal neurons, mouse cerebellar slices, and neuroblastoma cell lines, CB1Rs have been shown to block N-type Ca2+ channels (237–239). It has hypothesized, but only recently demonstrated, that the CB1R controls Ca2+ inflow to reduce the release of y-aminobutyric acid (GABA) in mouse hippocampus slices by altering the activity of presynaptic N-type Ca2+ channels (240). CB1R has been demonstrated to adversely regulate a variety of Ca2+ channel subtypes, including P/Q-type and R-type Ca2+ channels (241). But when CB1R complementary deoxyribonucleic acid (cDNA) is injected into transfected AtT-20 cells, Mus musculus nucleus accumbens slices and rat sympathetic neurons, the CB1R activates GIRK and triggers the activity of G-protein-coupled deeply changing potassium ion channels (242, 243).

Previous research has demonstrated that stimulation of the CB1R causes the extracellular signal-regulated kinase 1/2 (ERK1/2), c-Jun N-terminal kinase (JNK), mitogen-activated protein kinase (MAPK) and p38 signaling pathways, which have vital role in the regulation of cell cycle control, cell proliferation, and cell death to become active in a system that expresses the receptor endogenously or heterogeneously (235, 236, 244). The way that CB1R modulates MAPK signaling typically depends on the cell type and ligand (235). For instance, depending primarily on the microenvironment and stimulus type, CB1R-induced ERK1/2 activation can be mediated by G protein, β-arrestin, or phosphatidylinositol-3-kinases (PI3K) (245–247). Similar to this, CB1R stimulation has been shown to activate p38 in rat/mouse hippocampus slices, transfected Chinese hamster ovary (CHO-K1) cells, and human vascular endothelial cells (248). In transfected CHO-K1 cells, JNK activation has been demonstrated, and G proteins, PI3K, and the transduction was mediated by the reticular activating system (Ras) (249). Additionally, JNK initiation was seen in Neuro2A cells that express CB1R endogenously, which may be connected to CB1R-mediated neurite propagation (250).

The CB1R is able to communicate in a G protein-independent manner by linking with additional molecules such as -arrestin, in addition to the conventional G protein-dependent communication present with all G protein coupled receptors (GPCRs) (244). GPCR desensitization is primarily mediated by β -arrestin. β -arrestin attaches to the receptor after GRK phosphorylates it, starting the internalization process, during which β-arrestin may mediate signaling pathways (251). It has been demonstrated that β-arrestin 2-dependent desensitization of the CB1R occurs in a variety of settings (252, 253). According to research done in transfected human embryonic kidney cells (HEK-293), the timing of ERK1/2 phosphorylation in response to CB1R activation is controlled by β-arrestin 2-mediated desensitization but not by CB1R internalization (254). Additionally, follow-up investigations showed a beneficial relationship between the duration of CB1R association with -arrestin at the cell surface in a ligand-specific way and the degree of β-arrestin-mediated signaling (246). Studies with mice deficient in -arrestin 2 have indicated that this protein is crucial for controlling CB1R activity (255, 256). The CB1R expression in the -arrestin 2 knockout mice was similar, but they were more sensitive to THC, with improved antinociception and reduced tolerance (255, 256). In response to the CB1R allosteric modulator ORG27569. A recent study revealed that MAPK kinase ½, ERK1/2, and the proto-oncogene tyrosine-protein kinase Src are all phosphorylated by -arrestin 1, highlighting a signaling mechanism that is heavily reliant on stimuli (257).

In addition to MAPK signaling, the phosphatidylinositol 3-kinase/protein kinase B (PI3K/Akt) pathway also plays a significant role in regulating cell growth and death. The CB1R has been demonstrated to activate the PI3K/Akt pathway in Ratus ratus fundamental astrocytes, the Homo sapiens astrocyte cell line, and transfected CHO-K1 cells, which is in charge of the CB1R-induced protective role on cell survival (245). The PI3K/Akt pathway is used by rat oligodendrocyte progenitors to regulate cell differentiation and improve cell survival against food restriction (258, 259). Similar to this, HU-210, a selective CB1R agonist, protects against the neurotoxin (S)-amino-3-hydroxy-5-methyl-4-isoxazolepropionic acid in cultured rat cortical neurons by activating the PI3K/Akt pathway but not the MAPK pathways (260). In various brain areas, acute THC treatment in mice activated the PI3K/Akt pathway but not the ERK1/2 pathway (260). Recent research on huntingtin knock-in striatal neuronal cells showed that PI3K/Akt signalling increased the expression of brain-derived neurotrophic factor (BDNF), which allowed CB1R to defend neurons against excitotoxicity (261). Additionally, it has been demonstrated that CB1R-mediated PI3K/Akt activation influences oocyte maturation and embryonic development (262).

Treatment of Peripheral Blood Mononuclear Cells (PBMC) by THC or CBD significantly reduced the mitogen-induced synthesis of neopterin, a cellular immunity marker. However, the pretreatment of PBMC with nanomolar doses of THC or CBD increased the amount of Interferon‐gamma (IFN‐γ) secreted in response to phytohemagglutinin (PHA), micromolar dosages effectively reduced the amount of this pro-inflammatory cytokine produced as a result of activation (263). Additionally, the biphasic effects of THC and CBD were seen in the mitogen-induced breakdown of the tryptophan, which is mediated by indoleamine-2,3-dioxygenase, and is a crucial adaptive immune defense mechanism (263).

## 15 Challenges with use of cannabinoids in animals

Major challenges and limitations that may have an impact on the potential use of cannabinoids in animals are:

### 15.1 Cannabinoid’s stability and durability during storage, heating, and exposure to light and oxygen

Stability studies, a vital component of pharmaceutical research, enable the capacity to assess the therapeutic effects of an active pharmaceutical ingredient (API) or a finished pharmaceutical output while taking numerous environmental factors into account. Understanding CBD’s physical, chemical, and biological properties as well as information on its stability and shelf life is crucial to guarantee that it is utilized correctly in medicine. While Carbone et al. (264) gave an essentially comprehensive overview of THC degradation products. Cannabis resin and extract were shown to be extremely sensitive to oxygen-induced disintegration, light, and temperature (265). Layton et al. (266) concentrated more on the identification of degradation products generated by the aforementioned conditions, however because of the length of the experiment and the use of methanolic matrices, the results are not totally pharmaceutically acceptable.

It was important to discover an efficient, sensitive, and selective analytical approach for the detection and quantification of CBD and its potential degradation products in order to assess the impact of heat, humidity, oxygen access, matrix, and light. The literature mentions a few studies where cannabinoids were measured in cannabidiol-rich products using a combination of ultra-violent detection coupled with electrospray ionization tandem mass spectrometry (UV and MS/MS), while cannabinoids were analyzed in different matrices using isocratic and gradient elution profiles (267–269). It is challenging to separate cannabinoids under isocratic conditions because of their unique physical and chemical properties (268, 270). The results of the stability study on CBD powder were supported by an experiment on stability that examined how dried cannabis plant material would react to greater temperatures. They demonstrated that heat exposure at 37°C and 50°C results in a considerable loss of cannabinoids in the first 10 weeks, even if the CBD content in all of the stored materials remained largely steady without any evident deterioration for 100 weeks (271).

Because of the possibility for change when a by-product is saved in light exposure, photostability studies, which are necessary to determine CBD’s overall light sensitivity, are critical. Cannabinoids are least stable in photons, according to some scientists, although this also depends on other circumstances including the chemicals in which they are saved, temperature, O_2_ access, and many other aspects (272). THC and CBD were stable for 6 days when exposed to both naturally occurring and artificial if stored in both crude extract and solution for, indicating that light-exposed samples stored in different solvents degraded quicker than ones held in the dark. The key inference that can be made is that while prolonged exposure to light alone does not significantly alter the CBD concentration, light may hasten the process of degradation when paired with other factors like as the solvent employed, high temperature, and the presence of oxygen.

### 15.2 Problem in maintaining homogeneity in cannabinoids content in final products

During cannabis extraction operations, specific chemicals and solvents are routinely used, including propane, water, hydrocarbons, ethanol, butane, acetone, isopropanol, and hexane (273, 274). In addition to being employed by illegal extraction operations, these solvents are also used to lower production expenditure and retain terpenes that were already expended (275, 276). According to a current study of fifty-seven cannabis samples, more than 80 percent of the concentrates tested included residual solvents (277). This resulted from the use of chemicals in machine operations and product packaging when processing cannabis (278). Terpenes are being added to tinctures, vape oils, lotions, meals, and beverages by manufacturers of cannabis concentrates and derivative goods to improve flavor, assert health advantages, or recreate the original terpene profile that was lost during the cannabis extraction process. To modify the product’s viscosity and reduce production costs, medium-chain triglycerides, propylene glycol, or polyethylene glycol are also added to vape oil (279). Whether they are synthetic, botanical, or cannabis-derived, these additional terpenes represent another potential source of leftover solvents in cannabis-infused products. Additionally, throughout the vaping process, additional terpenes and thinning/cutting agents may collaborate or experience thermoxidative degradation to create, among other things, analytes used in residual solvent compliance testing (280). Although they fall outside the current compliance rules, residual solvents created in this way are nevertheless a significant public health concern. The bulk of published residual solvents test regulations refer to USP 467, the standard for pharmaceutical goods in the industry (280). The testing methodologies defined in USP 467 have been utilized for many years, and the usual solvents encountered in drug components, excipients, and final products were clearly recognized.

Analytical technologies are established to know potential of *Cannabis sativa* and its derivative products as pollutants. However, there would be a continual urge to develop the universal methodology to cannabis testing as more testing data are gathered, more proficiency testing programs are assessed. This would continue to support consumer safety and lead the development of laws and testing standards for goods derived from hemp. Consider potency as an illustration; it continues to be a key factor in the cannabis industry’s widespread consumer preference (280). Testing labs and their support services will continue to face challenges as the market for cannabis derivatives develops due to tighter regulatory oversight and an increase in the variety of matrices. Additionally, as other cannabinoids, such as 8-THC, come under regulatory oversight, new laboratory tools may be required to satisfy method specificity criteria. For the other test techniques mentioned in this article, comparable sets of difficulties exist (280).

It is also important to remember that secondary metabolites of interest in cannabis go beyond terpenes and cannabinoids. Flavonoids are one of several additional compounds of interest that could be used in cannabis testing (281). When these criteria become reality, the analytical testing community will need to use what it has learned about cannabis’ difficulties as a matrix to build appropriate testing procedures. Fortunately, significant advancements have lately been achieved in our comprehension of the constraints placed on analytical testing of cannabis and its byproducts. This is a direct result of regulatory changes that have allowed cannabis science to enter the commercial market. When there are monetary benefits, there will be increase in effective testing regimes, though not beyond increasing pains because of delay in the accessibility of the crucial testing framework (280).

### 15.3 Lack of global standardized regulation on the use of hemp and cannabinoids

There are many CBD products available, some of which are marketed as medicines for various conditions as well as other items that are produced and disseminated without regulations and frequently have unproven ingredients (282). The U.S. Food and Drug Administration has sent manufacturers 2 major series of caution notifications for false medical assertions (explaining health assistance and wellbeing without any supporting data) and false production claims (marketing products as having a certain concentration of CBD when testing shows that it doesn’t (282).

## 16 Prospects of using cannabinoids as potential feed additive in animals

The hemp plant can be used to produce a variety of feed materials, including hemp seed meal/cake, *Cannabis sativa* seed oil, and the entire herb (including *Cannabis sativa* seed shives, fresh or dried). *Cannabis sativa* flour (ground dried *Cannabis sativa* leaves) and *Cannabis sativa* protein segregates (from seeds) are other goods. All animal species could utilize hemp seed and hemp seed cake as feed, and the EFSA set up most incorporation values in the whole feed for each species, such as 3 to 7 percent for chickens, 2 to 5 percent for *Sus domesticus*, 5 percent for cattle, and 5 percent for aquatic species for hemp *Cannabis sativa*. Additionally, feed conversion ratio studies show that hemp and its derivatives can be used as a suitable supply of vital lipids and crude protein for cattle diets (283).

Due to its high fiber content, the entire hemp plant, including the stem and leaves, is regarded as an acceptable source of food for ruminants (and horses). All species of animals can be fed on *Cannabis sativa* seed and *Cannabis sativa* seed cake. When introducing such goods into the total feed, a number of particular species constraints (fiber for hens, FA for *Sus domesticus*, etc.) should take into account. Hemp seed contains a part of rumen-indigestible protein, which is favorable for ruminants (97). According to information from feeding trials, hemp seed cake might be utilized up to 20 percent in the diets of laying hens; it is therefore determined that no more than 10 percent can be used in the diets of hens for weight gain. Although there is null information on pigs, it is anticipated that 10 percent *Cannabis sativa* seed cake and 5 percent *Cannabis sativa* seed could be utilized in pig complete feed. According to data, dairy cows can get a total mixed ration that contains 14 percent hemp seed cake. Comparable research on the upbringing of calves and fattening of cattle revealed that one to 1.4 kg of *Cannabis sativa* seed cake could be given per day (97).

Because hemp products are extremely limited in terms of quantity and price, the maximum integration rates in the formulation of compound feeding stuffs are probably lower than the aforementioned values; as a result, it is difficult to determine what they would be (47). The following maximal assimilation values in feed could be accepted in normal manufacturing and production if considerable volumes of hemp products are locally accessible: Pigs 2 to 5 percent hemp seed/hemp seed cake; ruminants’ 5 percent in the routine daily feed; fish 5 percent; poultry for increase in weight 3 percent and laying poultry 5 to 7 percent. It must be highlighted that these values or numbers cannot be viewed as cumulative as the concurrent application of hemp by products would vastly outweigh available resources. Entire herb (or portions of it, like leaves) may be eaten as forage by ruminant.

## 17 Conclusion and future prospects

The present study concluded that hemp or its cannabinoids possess excellent potential to modulate health and performance of animals. The active cannabinoids have shown excellent antioxidant and immune-modulatory activities making them promising dietary additives especially under oxidative stress and disease conditions, respectively. Besides the leaves and seeds of Cannabis sativus, its by-products (oil cakes etc.) also are being used in animal feeds as supplements. Different treatment strategies (e.g ensiling or solid-state fermentation) have been used to avoid some adverse effects of Cannabis feeding on animals. However, further studies are required to optimize best feeding levels of hemp and cannabidiols in animal diets to get desirable outputs in terms of better health and production of animals. Moreover, in-depth research will be needed to understand the therapeutic efficacy of cannabinoids on various health aspects in diverse animal species, examining optimal dosage and administration methods, exploring potential side effects and safety profiles, and delving into the underlying mechanisms of cannabinoid action. Additionally, long-term impacts and feasibility of incorporating cannabinoids into veterinary practices could be the crucial aspects for future research.

## Author contributions

F-UH: Conceptualization, Writing – original draft. CL: Software, Writing – review & editing. MM: Writing – review & editing. RB: Writing – review & editing, Data curation, Visualization. MA: Writing – review & editing, Validation. FS: Writing – review & editing. FC: Writing – review & editing, Investigation, Methodology. YL: Writing – review & editing, Formal Analysis. JZ: Writing – review & editing, Software, Validation. PS: Writing – review & editing, Formal Analysis. BL: Writing – review & editing, Investigation, Methodology. QL: Project administration, Supervision, Writing – review & editing.
